# Are Sex-Specific Cutoffs Needed With a Next-Generation Urine Tenofovir Lateral Flow Assay for Antiretroviral Adherence Monitoring?

**DOI:** 10.1093/ofid/ofaf512

**Published:** 2025-08-21

**Authors:** Xin Niu, Derin Sevenler, Sandy Dossantos, Mehmet Toner, Rebecca Sandlin, Oraphan Siriprakaisil, Pra-ornsuda Sukrakanchana, Tim R Cressey, Paul K Drain

**Affiliations:** Department of Epidemiology, University of Washington, Seattle, Washington, USA; International Clinical Research Center, Department of Global Health, University of Washington, Seattle, Washington, USA; Center for Engineering in Medicine & Surgery, Massachusetts General Hospital, Charlestown, Massachusetts, USA; Harvard Medical School, Boston, Massachusetts, USA; Center for Engineering in Medicine & Surgery, Massachusetts General Hospital, Charlestown, Massachusetts, USA; Center for Engineering in Medicine & Surgery, Massachusetts General Hospital, Charlestown, Massachusetts, USA; Harvard Medical School, Boston, Massachusetts, USA; Center for Engineering in Medicine & Surgery, Massachusetts General Hospital, Charlestown, Massachusetts, USA; Harvard Medical School, Boston, Massachusetts, USA; Sanpatong Hospital, Chiang Mai, Thailand; AMS-PHPT Research Collaboration, Faculty of Associated Medical Sciences, Chiang Mai University, Chiang Mai, Thailand; AMS-PHPT Research Collaboration, Faculty of Associated Medical Sciences, Chiang Mai University, Chiang Mai, Thailand; Department of Pharmacology and Therapeutics, University of Liverpool, Liverpool, UK; Department of Epidemiology, University of Washington, Seattle, Washington, USA; International Clinical Research Center, Department of Global Health, University of Washington, Seattle, Washington, USA; School of Medicine, University of Washington, Seattle, Washington, USA

**Keywords:** adherence, HIV, PrEP, tenofovir, urine

## Abstract

Accurate point-of-care tools are needed to detect early nonadherence to daily HIV regimens and support timely transitions to long-acting options. Emerging evidence suggests that females may require higher adherence than males to achieve equivalent protection. Our next-generation urine tenofovir assay showed high accuracy across sexes but lower urine drug levels among female participants.

Despite the increasing availability of effective oral antiretroviral treatment (ART) and preexposure prophylaxis (PrEP), an estimated 1.3 million new human immunodeficiency virus (HIV) infections were reported globally in 2023 [1]. Although adherence counseling is a standard component of ART/PrEP visits, inconsistent drug adherence remains a major global challenge, with only 65% of people living with HIV on ART achieving virologic suppression and 29% of PrEP users maintaining optimal daily dosing [2, 3]. To meet the 95-95-95 UNAIDS targets, substantial improvements in both ART and PrEP adherence are needed. While long-acting injectables have expanded options for individuals who struggle with daily oral regimens, effective and scalable tools for monitoring adherence remain limited. Such tools are essential for identifying individuals with adherence challenges and facilitating early transitions to long-acting injectables when appropriate.

The World Health Organization guidelines recommend oral tenofovir (TFV)–based regimens for first-line ART and PrEP. Point-of-care tests measuring TFV levels in the urine may allow for better monitoring and interventions to enhance adherence [4]. A urine TFV lateral flow assay (LFA) has been developed and, using a TFV threshold of 1500 ng/mL, has demonstrated high specificity to detect PrEP nonadherence (accurately classifying 98% of users who took a dose of TVF disoproxil fumarate [TDF]–based oral PrEP within 24 hours as adherent) [5]. Published data have indicated a sex-based difference in drug concentrations despite similar levels of adherence [6, 7]. Women may also require higher adherence than men to achieve equivalent protection [8], which suggests that sex-specific cutoffs for urine TFV LFAs may be necessary. Further efforts are also needed to improve the sensitivity of this urine LFA in detecting nonadherence during the 24–96-hour window since the last dose.

We developed a next-generation urine TFV LFA that does not have a predetermined threshold for urine TFV concentration, and we evaluated the quantified readouts (optical readings and visual scores) using urine specimens from a directly observed PrEP adherence randomized trial [9]. This study aimed to (1) determine whether the LFA-quantified results differed between female and male study participants with the same observed adherence levels, (2) evaluate the diagnostic performance of the LFA for detecting PrEP nonadherence by sex, and (3) assess the ability of the LFA to detect nonadherence with a more stringent definition that may reduce white-coat dosing (the practice of taking dose shortly before a clinic visit—typically within the 24 hours before drug level testing—to appear adherent).

## METHODS

We conducted a randomized controlled pharmacokinetic trial of adults receiving varying adherence levels of oral PrEP in Thailand, with directly observed therapy for each drug ingestion (ClinicalTrials.gov NCT0301260) [10]. To approximate groups with low, moderate, and perfect adherence, healthy adults were randomized to receive either 2, 4, or 7 doses/week of TDF 300 mg/emtricitabine (FTC) 200 mg for 6 weeks. We recorded the time since last TDF/FTC pill for each urine sample collected.

We tested the urine samples using a next-generation urine TFV LFA with 3 quantified readouts [7]. Since this is a competitive assay, higher concentrations of urine TFV will prevent more TFV-specific antibody to conjugate and appear at the LFA test line. Therefore, higher drug concentrations in urine result in weaker color intensity on the test line. Two trained laboratory technicians performed independent visual scoring of the LFA test line from each deidentified sample against the same color intensity reference card, which had a graduated scale from 0 (not visible) to 5 (very dark). Averaged visual scores from 2 laboratory technicians were used to account for variations between users. Quantified LFA optical readings were also obtained using a commercial strip reader (Axxin Ax-2x-S) for the peak color intensity of the test line. Ratios of the test versus control line peak color intensity readings were also calculated as a comprehensive optical readout. Averaged visual scores and optical readings for the same sample were independently obtained, with each assessment conducted without knowledge of the other.

We compared the LFA readouts by sex among adherent samples with a last dose taken in the prior day. We performed generalized estimating equations with unstructured correlations to assess the effect of sex on the LFA readouts. The time in hours since the last dose was included as the time variable, while the randomized adherence arm was adjusted as a confounder.

We calculated overall and sex-specific sensitivities and specificities for recent nonadherence (defined as not having taken a dose within the prior 24 hours) using receiver operating characteristic (ROC) analysis of clustered data. We computed the 95% confidence intervals (CIs) with stratified bootstrap. Optimal cutoffs were determined based on the Youden index. To reduce potential impact from white-coat dosing, ROC analyses were also performed for recent nonadherence with a more stringent definition (not having taken 2 consecutive daily doses within the prior 48 hours). The assumption was that white-coat dosing would be rare among those who have taken 2 consecutive daily doses within the prior 48 hours.

Written consent was obtained from the study participants. The design of the study was approved by ethics committees at the Institute for the Development of Human Research Protections at the Medical Sciences Department, Thai Ministry of Public Health, the ethics committee of Sanpatong Hospital, the Faculty of Associated Medical Sciences, Chiang Mai University, and the University of Washington Institutional Review Board.

## RESULTS

### Participant/Specimen Characteristics

A total of 586 urine specimens were evaluated, from 28 participants (43% female) with a median age (interquartile range) of 33 (28–40) years (Supplementary Table 1). The perfect adherence group had more female participants (6 of 9 [67%]) than the low (4 of 9 [44%]) and moderate (2 of 10 [20%]) adherence groups. Seventy-six percent of urine samples (447 of 586) had a time since last dose of >24 hours and were thus classified as “nonadherent.” Using the more stringent definition of nonadherence, 453 urine samples were collected when the participants did not take 2 consecutive daily doses in the prior 48 hours.

### Sex Differences in Descriptive Statistics

Among 139 urine samples with a time since last dose of ≤24 hours, the medians of the optical readings for the test line were 385 and 340 among female and male participants, respectively. Compared with male participants, we also observed 0.5-unit higher median averaged visual score for urine samples from female participants (Supplementary Table 2). Among samples with a time since last dose of 10–24 hours, scatterplots showed female participants having higher values than male participants for all 3 LFA readouts (indicating lower urine TFV levels) (Supplementary Figure 1*A*–1*C*). Overall, male participants had 33% lower (*P* < .01) optical readings and a 1.82-unit lower (*P* < .01) averaged visual score of the LFA test line, compared with female participants (Supplementary Table 3).

### ROC Analysis for the Total Population

To detect “no dosing in the prior day,” the LFA demonstrated the highest sensitivity (93% [95% CI, 91%–95%]) with a test–control line color intensity ratio of >0.095, while maintaining a high specificity (96% [93%–99%]) in the total population (Table 1). By using the more stringent definition for recent nonadherence, we observed similarly high specificities across all LFA readouts but lower sensitivity from the averaged visual score (78% [95% CI, 74%–81%]) compared with the test–control line optical reading ratio (92% [89%–94%]).

**Table 1.** Diagnostic Performance of a Next-Generation Tenofovir Lateral Flow Assay for Nonadherence Detection

Nonadherence	Average Visual Score >1.25	Test Line Peak Color Intensity >705	Test–Control Line Peak Color Intensity Ratio >0.095
No dosing in prior day			
All participants (N = 586)			
Sensitivity (95% CI), %; LR+	79 (75–82); 16	86 (83–89); 43	93 (91–95); 23
Specificity (95% CI), %; LR−	95 (91–95); 0.22	98 (95–100); 0.14	96 (93–99); 0.07
Male participants (n = 341)			
Sensitivity (95% CI), %; LR+	76 (71–81); 25	86 (82–91); NA	93 (90–96); 93
Specificity (95% CI), %; LR−	97 (93–100); 0.25	100 (100–100); 0.14	99 (96–100); 0.07
Female participants (n = 245)			
Sensitivity (95% CI), %; LR+	82 (77–88); 10.3	86 (81–91); 17	93 (89–96); 16
Specificity (95% CI), %; LR−	92 (86–98); 0.09	95 (89–100); 0.15	94 (88–98); 0.07
No 2 consecutive daily doses in the 2 days (all participants; N = 586)			
Sensitivity (95% CI), %; LR+	78 (74–81); 16	85 (82–88); 85	92 (89–94); 23
Specificity (95% CI), %; LR−	95 (91–95); 0.23	99 (96–100); 0.15	96 (93–99); 0.08

Abbreviations: CI, confidence interval; LR+, positive likelihood ratio; LR−, negative likelihood ratio; NA, not applicable.

### Sex-Specific ROC Analysis

The LFA showed minor differences for diagnostic sensitivity and specificity among male and female study participants (Table 1). With the same optimal cutoffs (>0.095) for the test–control line optical reading ratios, we found similar sensitivities (93% vs 93%) and specificity (99% vs 94%) among male versus female participants. Comparable sensitivities (86% vs 86%) and specificities (100% vs 95%) by sex were observed when using the same cutoff (>705) for the test line optical reading. However, the sensitivity was slightly better among female than among male participants (82% vs 76%, respectively) when using the same cutoff (>1.25) for the averaged visual score.

## DISCUSSION

In a randomized directly observed oral PrEP adherence trial, we found higher LFA readouts (indicating lower urine TFV levels) among female than among male participants, using both the averaged visual score and the optical reading. However, these differences had a minimal impact on diagnostic accuracy of a next-generation LFA. The best results were obtained using the ratio of the test and control line optical readings. The next-generation LFA also demonstrated enhanced sensitivity (92%) while preserving a high specificity (96%) in identifying nonadherence (including white-coat dosing) to TDF-based oral PrEP.

Sex differences in antiretroviral drug concentrations and effectiveness have been described elsewhere. Clinical trials of directly observed PrEP have revealed higher intracellular TFV diphosphate (TFV-DP) concentrations in dried blood spots among women than among men with the same adherence levels [6, 10]; Pharmacokinetic studies have reported lower drug concentrations in vaginal than in rectal tissue [11, 12]. Compared with that required in men who have sex with men, the minimum PrEP adherence required to achieve sufficient HIV protection has not been well characterized for women. The HIV Prevention Trial Network 083 and 084 studies indicated that women may require higher adherence to achieve the same level of risk reduction as men [6]. However, a recent pooled analysis estimated a low HIV incidence (0.13/100 person-years) for women with 4–6 doses/week, suggesting PrEP adherence benchmarks comparable to those established for men who have sex with men [13]. However, due to diverse characteristics across different adherence groups, the pooled analysis did not assess relative risk reduction or PrEP efficacy for different gradients of adherence. These findings highlight the importance of understanding sex differences in the measurement of drug concentrations for monitoring antiretroviral adherence.

Consistent with prior studies, our study also observed sex differences in PrEP drug concentrations. However, unlike the higher intracellular TFV-DP in female study participants, we found lower urine TFV (as indicated by higher LFA readouts) among female than among male participants who took a dose in the prior day. These opposing sex differences can be explained by faster drug clearance among men, leading to higher levels of TFV in urine but lower intracellular TFV-DP accumulation than in women. Nevertheless, this sex difference had a minimum impact on the diagnostic performance for nonadherence detection. The next-generation LFA also represents an advancement in sensitivity for detecting recent nonadherence (including white-coat dosing). Even in resource-limited areas, where optical readers may not be readily available, averaged visual scores from the LFA accurately classified 95% of PrEP users who took 2 consecutive daily doses in the prior 2 days as adherent. To further reduce cost and turnaround time, ongoing efforts are directed toward developing and validating smartphone applications for image analysis and self-effectiveness monitoring.

In summary, while female study participants had higher LFA readouts (indicating lower urine TFV levels) than male participants, sex-specific cutoffs for a next-generation TFV LFA may not be necessary to measure TDF-based antiretroviral drug adherence. The next-generation TFV LFA achieved high accuracy for detecting recent nonadherence and holds promise for applications in real-time ART and PrEP adherence monitoring.

## Supplementary Material

ofaf512_Supplementary_Data

## Supplementary Data

Supplementary materials are available at *Open Forum Infectious Diseases* online. Consisting of data provided by the authors to benefit the reader, the posted materials are not copyedited and are the sole responsibility of the authors, so questions or comments should be addressed to the corresponding author.
