# Supplementary material for: Are Sex-Specific Cutoffs Needed With a Next-Generation Urine Tenofovir Lateral Flow Assay for Antiretroviral Adherence Monitoring?
Source: Open Forum Infect Dis. 2025 Aug 21;12(9):ofaf512. doi: 10.1093/ofid/ofaf512 (PMC12415330; doi:10.1093/ofid/ofaf512)
Supplement: ofaf512_Supplementary_Data [file ofaf512_supplementary_data.docx]

**Supplementary Materials**

**Table S1**. Baseline Participant Characteristics by Randomized Adherence Arm^*^

|  | Low (2 doses/week) |  | Moderate (4 doses/week) |  | Perfect (7 doses/week) |
| --- | --- | --- | --- | --- | --- |
| Baseline Characteristics | (N=9) |  | (N=10) |  | (N=9) |
| Age, years | 38(27-40) |  | 32(28-33) |  | 34(31-39) |
| Female, N(%) | 4(44%) |  | 2(20%) |  | 6(67%) |
| Body Mass Index, kg/m2 | 23.1(20.2-28.4) |  | 24.0(22.3-25.0) |  | 20.2(19.1-24.3) |
| Hemoglobin, g/dL | 14.7(13.3-15.0) |  | 14.5(14.4-15.1) |  | 12.4(11.9-13.2) |
| Hematocrit, % | 43.0%(39.2%-45.1%) |  | 43.8%(41.9%-45.2%) |  | 38.0%(36.2%-39.3%) |
| eGFR, mL/min | 108(102-115) |  | 124(101-132) |  | 91(87-108) |
| BUN, mg/dL | 10.5(10.2-12.3) |  | 10.4(8.7-13.0) |  | 9.3(9.0-11.0) |
| Creatinine (plasma), mg/dL | 0.86(0.75-1.01) |  | 0.84(0.78-.96) |  | 0.73(0.68-0.81) |
| Total bilirubin, mg/dL | 0.58(0.47-0.79) |  | 0.84(0.55-1.07) |  | 0.68(0.35-0.76) |

*Median(Interquartile range) if not specified

**Table S2**. Next-Gen LFA Readouts among Urine Samples with Time Since Last Dose ≤ 24h (One Dose in the Prior Day) Stratified by Sex

| Urine Samples  (one dose in the prior day) | **Male**  **(N=75)** | | **Female**  **(N=64)** | |
| --- | --- | --- | --- | --- |
|  | Median  (interquartile range) | Mean  (min-max) | Median  (interquartile range) | Mean  (min-max) |
| Optical Reading of the Test Line Peak Color Intensity | 340  (270-452) | 359  (160-680) | 385  (290-568) | 432  (160-1120) |
| Ratio of the Optical Readings of the Test/Control Line Peak Color Intensity | 0.06  (0.05-0.07) | 0.06  (0.03-0.10) | 0.06  (0.05-0.08) | 0.06  (0.02-0.15) |
| Averaged Visual Score of the Test Line | 0  (0-0.5) | 0.29  (0-2.0) | 0.5  (0-1.0) | 0.49  (0-2.0) |

**Figure S1a**: Scatterplot of Log_10_ Optical Readings of the Test Line Peak Color Intensity by Hours Since Last Dose


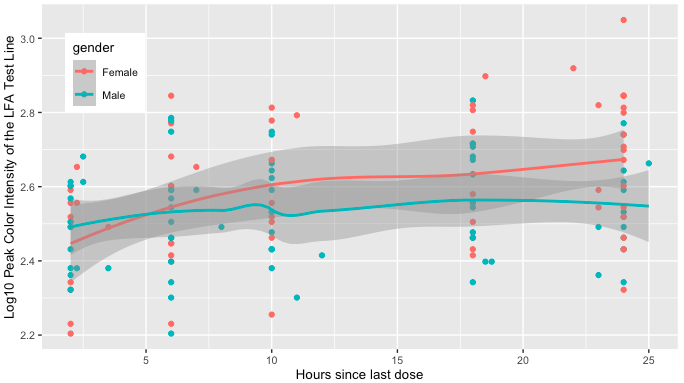


**Figure S1b**: Scatterplot of Ratio of the Optical Readings of the Test/Control Line Peak Color Intensity by Hours Since Last Dose


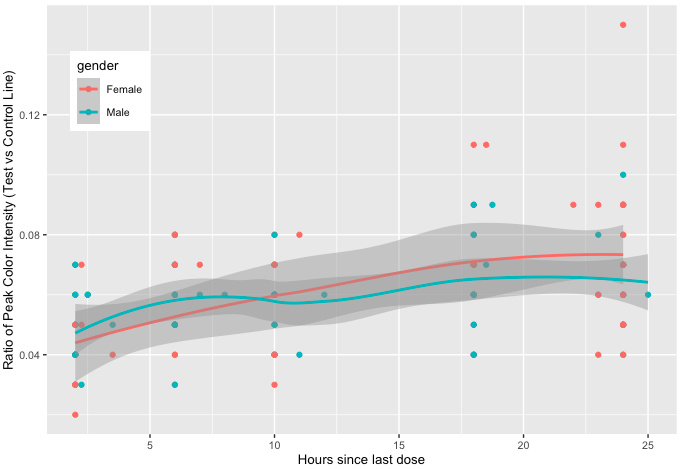


**Figure S1c**: Scatterplot of the Averaged Visual Score of the Test Line by Hours Since Last Dose


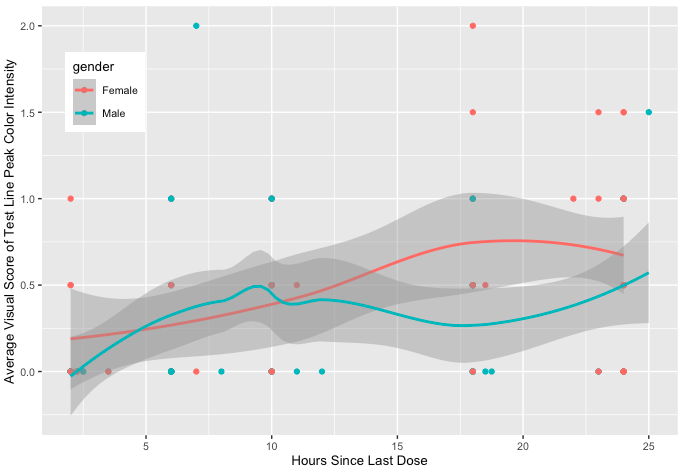


**Table S3**. GEE Results for LFA Readouts among Urine Samples with Time Since Last Dose ≤ 24h^*^

|  | Log 10 Test Line Peak Color Intensity | Test/Control Line Peak Color Intensity Ratio | Averaged Visual Scores |
| --- | --- | --- | --- |
| Male (vs Female) | -**0.17**  **(-0.258 to -0.082)** | 0.04  (-0.025 to 0.11) | **-1.82**  **(-2.70 to -0.94)** |
| One additional hour since last dose | 0.0034  (-0.0001 to 0.0069) | 0.0017  (0.0012 to 0.0022) | **-0.31**  **(-0.47 to -0.15)** |

*GEE (Generalized Estimating Equations); (95% Confidence Interval) if not specified
